# Efficacy of Transcranial Direct Current Stimulation (tDCS) on Balance and Gait in Multiple Sclerosis Patients: A Machine Learning Approach

**DOI:** 10.3390/jcm11123505

**Published:** 2022-06-17

**Authors:** Nicola Marotta, Alessandro de Sire, Cinzia Marinaro, Lucrezia Moggio, Maria Teresa Inzitari, Ilaria Russo, Anna Tasselli, Teresa Paolucci, Paola Valentino, Antonio Ammendolia

**Affiliations:** 1 Department of Medical and Surgical Sciences, University of Catanzaro “Magna Graecia”, Via Tommaso Campanella 115, 88100 Catanzaro, Italy; nicola.marotta@unicz.it (N.M.); cinziamarinaro83@gmail.com (C.M.); lucrezia.moggio@gmail.com (L.M.); inzitari@unicz.it (M.T.I.); ilaria.russo003@gmail.com (I.R.); annatasselli@gmail.com (A.T.); ammendolia@unicz.it (A.A.); 2 Physical Medicine and Rehabilitation, Department of Oral, Medical and Biotechnological Sciences, University of Gabriele D’Annunzio of Chieti, 66100 Chieti, Italy; teresa.paolucci@unich.it; 3 Institute of Neurology, University of Catanzaro “Magna Graecia”, Viale Europa, 88100 Catanzaro, Italy; p.vale@unicz.it

**Keywords:** multiple sclerosis, tDCS, neurorehabilitation, rehabilitation, gait analysis, mobility, machine learning, multiple factor analysis

## Abstract

Transcranial direct current stimulation (tDCS) has emerged as an appealing rehabilitative approach to improve brain function, with promising data on gait and balance in people with multiple sclerosis (MS). However, single variable weights have not yet been adequately assessed. Hence, the aim of this pilot randomized controlled trial was to evaluate the tDCS effects on balance and gait in patients with MS through a machine learning approach. In this pilot randomized controlled trial (RCT), we included people with relapsing–remitting MS and an Expanded Disability Status Scale >1 and <5 that were randomly allocated to two groups—a study group, undergoing a 10-session anodal motor cortex tDCS, and a control group, undergoing a sham treatment. Both groups underwent a specific balance and gait rehabilitative program. We assessed as outcome measures the Berg Balance Scale (BBS), Fall Risk Index and timed up-and-go and 6-min-walking tests at baseline (T0), the end of intervention (T1) and 4 (T2) and 6 weeks after the intervention (T3) with an inertial motion unit. At each time point, we performed a multiple factor analysis through a machine learning approach to allow the analysis of the influence of the balance and gait variables, grouping the participants based on the results. Seventeen MS patients (aged 40.6 ± 14.4 years), 9 in the study group and 8 in the sham group, were included. We reported a significant repeated measures difference between groups for distances covered (6MWT (meters), *p* < 0.03). At T1, we showed a significant increase in distance (m) with a mean difference (MD) of 37.0 [−59.0, 17.0] (*p* = 0.003), and in BBS with a MD of 2.0 [−4.0, 3.0] (*p* = 0.03). At T2, these improvements did not seem to be significantly maintained; however, considering the machine learning analysis, the Silhouette Index of 0.34, with a low cluster overlap trend, confirmed the possible short-term effects (T2), even at 6 weeks. Therefore, this pilot RCT showed that tDCS may provide non-sustained improvements in gait and balance in MS patients. In this scenario, machine learning could suggest evidence of prolonged beneficial effects.

## 1. Introduction

Multiple sclerosis (MS) is a chronic neurodegenerative disease characterized by the presence of demyelinating lesions affecting the central nervous system [1]. With a predominantly young age of onset, MS is currently the leading cause of disability in young–middle-aged adults in the developed world [2]. MS is heterogeneous in both its symptomatic presentation and clinical progression, with motor, sensory and cognitive impairments frequently observed in varying magnitudes [3]. It is estimated that 40% of MS patients show gait impairment, worsening over time and interfering with daily activities [4,5,6]. Additionally, 60–70% of people with MS report problems with balance and an increased risk of falls, with a consequent disability [7,8,9,10].

Walking changes in MS include reduced gait speed, step length, cadence and joint motion and impaired walking endurance, as well as impaired coordination and increased metabolic cost of walking [11,12,13]. Gait impairment in MS might be due to cerebellar or sensory ataxia, spasticity, weakness and apraxia, including cognitive impairments, and vision disorders from optic atrophy could also contribute [14]. These impairments are most commonly assessed with performance measures such as the 6-min walk test (6MWT) and the timed up-and-go (TUG) tests, reflecting walking endurance and speed [15]. MS can also result in impairments in vestibular function, proprioception, vision and coordination, as well as in the integration of these functions, thereby leading to balance disorders [7,8,9,10]. A reduced ability to maintain position, slowed regulation of movement within the limits of stability, reduced adaptive responses to changes in body position and external perturbations represent the elements that best characterize balance disturbances in MS [16].

The most effective non-pharmacological approach to manage walking impairment and improve balance is the practice of physical exercise [17,18]. The rehabilitation of balance and gait deficits usually relies on the principles of neuroplasticity and motor learning strategies [19]. It is widely demonstrated that the rehabilitation could exploit the individual patient’s potential to restore nervous functions beyond the spontaneous mechanisms of recovery from MS-related damage, regardless of the disability level and tissue damage [20,21,22,23,24]. The goal of rehabilitation interventions is to minimize motor impairments while facilitating the activation of neural pathways in order to achieve the long-term recovery of walking [20,25]. While traditional rehabilitation techniques remain the benchmark, new technologies are emerging, allowing improved management of disabling symptoms [26,27,28,29].

Recently, the role of transcranial direct current stimulation (tDCS) in movement disorders has been taking on intriguing perspectives in dystonia, tremor and Parkinson’s disease, as well as degenerative cerebellar ataxia [30]. Among the non-invasive neurostimulation techniques, tDCS represents the predominant approach to motor and non-motor symptoms in MS [31]. It is a neurorehabilitation technique able to modulate cortical excitability and cortico-spinal circuits, inducing a subthreshold modification of neuronal resting membrane potentials [32]. Classically, anodal tDCS increases cortical excitability, whereas cathodal tDCS reduces it, both leading to long-lasting changes in neuronal activities of specific brain networks [33].

Anodal tDCS determines improvements in gait parameters both during and after stimulation in people with MS [34,35]. Applied to the primary motor cortex (M1), it ameliorates motor performance, leading to synergistic effects when combined with a conventional rehabilitation treatment [36,37]. Nevertheless, the exact influence of tDCS on gait in MS patients is mixed, being influenced by the degree of disability, the number of stimulation sessions and the type of parameters measured to define its effects [35,38]. Patients with MS with a low EDSS appear to benefit most from a combined treatment of neurostimulation and aerobic exercise and of a number of stimulation sessions that should be no less than 10 sessions [34,36,39]. Workman, et al. [40] demonstrated that stimulation during the 6MWT, which is a clinical measure of gait impairment for MS patients, significantly increased walking speed. Pilloni, et al. [34] showed that even a single session of anodal tDCS to the M1 during aerobic exercise was unable to improve gait speed and distance walked in 2 min at a 4-week follow-up [36].

The difficulty in analyzing literature data increases when comparing the effects on gait parameters. These are often based on subjective parameters, i.e., based on self-assessment questionnaires of gait quality, or on parameters that investigate only few aspects of the complex system that governs walking (short distances, long distances, posture maintenance) [41].

In the management of motor symptoms using tDCS, the main debate concerns the duration of the effects induced by the tDCS. Several studies conducted on stroke, cerebral palsy and Parkinson’s disease have found benefits on motor outcomes that are continuous and durable after repeated tDCS treatments [42,43,44]. On the other hand, the literature on MS is inconsistent, with significant data on short-term effects obtained via stimulation during motor task performance and prolonged effects obtained only by assessing motor task performance after stimulation [40].

In recent years, machine learning studies have been successfully improved its diagnostic capability in a wide range of medical applications, also being enhanced by support vector machines (SVM) [45,46,47,48]. Such relatively simple representatives of machine learning algorithms perform quite well when extracting the most relevant information from complex data, providing a more sophisticated approach to classification problems by often mimicking neural networks [20,49]. Machine learning methods, such as k-nearest neighbors, SVM and neural networks, were developed to identify, collect and evaluate pathological gait patterns [50]. Piryonesi, et al. demonstrated that machine processing can predict falls and injuries in MS patients using decision trees and gradient boosted trees [51].

In this scenario, the multiple factor analysis (MFA) is a multivariant technique in statistics that allows the analysis of several groups of continuous variables of different natures, allowing the clustering of individuals via a machine learning model [52,53]. The MFA adopts a the geometric approach based on a set of variables, vectorizing the inertia of each factor on the Cartesian axes [54].

To date, although the data concerning the use of tDCS in the treatment of motor symptoms are complex, tDCS appears to be one of the most applied technologies with an interesting implication in terms of balance alterations. Indeed, walking is a complex task involving the cooperation of several bodily functional systems, including postural control, on which the reference literature on neurostimulation seems not to provide exhaustive information.

To the best of our knowledge, this is the first study evaluating the impact of tDCS in MS patients with a combination of balance and locomotion parameters using a cluster analysis. Our hypothesis is to consider improvements in the functional parameters of balance and gait by stimulating the motor area, and confirming this with a cluster analysis.

Therefore, in such a complex scenario, the objective of the present study is to evaluate the tDCS effects over time on balance and gait in patients with relapsing–remitting MS by measuring single variable weights through a machine learning approach.

## 2. Materials and Methods

### 2.1. Study Design

This is a pilot randomized control trial (RCT) with a double-blind, sham-controlled design of tDCS therapy paired with therapeutic exercise for gait and balance performance. Ethical approval was obtained from the Institutional Review Board Committee (number 303/2019). The study was performed in accordance with the Ethical Principles for Medical Research Involving Human Subjects outlined in the Declaration of Helsinki. All participants were fully informed about all experimental procedures and signed a written informed consent form prior to participation.

### 2.2. Participants

Relapsing–remitting MS patients were recruited from the Outpatient Physical and Rehabilitative Unit of the University Hospital “Mater Domini” of Catanzaro, Italy. Inclusion criteria were: (1) diagnosis of relapsing–remitting MS according to the McDonald Criteria [55]; (2) 18–70 years of age; (3) an Expanded Disability Status Scale (EDSS) >1 and <5. We excluded patients with the following characteristics: (1) severe cognitive impairment (Mini-Mental Status Examination <24); (2) changes in disease-modifying medications within the last 21 days; (3) documented history of seizures and brain aneurysms; (4) metal prostheses in the skull or hearing aids; (5) consumption of medications that might influence neuroplasticity (i.e., benzodiazepines, anti-cholinergic medication, selective serotonin reuptake inhibitors, GABAergic medications).

### 2.3. Intervention

After the enrollment, all study participants were divided through a randomization scheme with 1:1 allocation into 2 groups: the experimental group, undergoing tDCS and physical therapy, and the control group, undergoing a sham tDCS and physical therapy.

In more detail, the interventional protocol was structured in a daily tDCS session with active or simulated stimulation lasting 20 min for 5 days a week for two weeks (10 sessions in total). The tDCS was delivered with a battery-powered electrical stimulator (Brainstim, EMS s.r.l., Bologna, Italy) connected to a pair of saline-soaked 25-cm^2^ synthetic surface sponge electrodes placed on the scalp. The sponges were soaked in saline solution until saturation. An M1-SO electrode montage was applied with the positive electrode (i.e., anode) over C3 (M1 cortex) and the negative electrode (i.e., cathode) over Fp2 (supraorbital margin) according to the 10–20 EEG system [32]. The left or right position of the anode electrode was determined according to the side of the body that was most affected. In the experimental group, the active tDCS condition consisted of 20 min of continuous stimulation at a maximum intensity of 2.0 mA. At the beginning of the stimulation, the current was increased over a 30 s period. Participants were instructed to notify the investigator if they felt any uncomfortable sensations arising from the stimulation. At the end of the session, the current was automatically ramped down to 0.0 mA over a 30 s period. On the other hand, in the control group, the device was programmed without the current being delivered during the session.

Both groups underwent conventional physical therapy at the end of each tDCS session, performing 30 min of balance training followed by 30 min of resistance training of the lower extremities. Specific balance programs were performed and consisted of proprioceptive, static and dynamic balance exercises with a computerized board (Balance Board, Biodex Medical Systems, Shirley, NY, USA) [56] under the supervision of an experienced physical therapist. Later, patients performed resistance training of the lower extremities that included combined aerobic and resistance exercises, consisting of 5 to 10 repetitions at 60% of predicted 1-RM for lower limb exercises, including knee flexion and extension, plantarflexion, trunk flexion and trunk extension in that order every time. Exercises were performed at a self-selected, comfortable pace with at least 1 min of rest between exercises [57]. Lastly, after a 5-min pause the program was concluded with a 3-min training with treadmill system (RTM 600 Biodex Medical System, Shirley, NY, USA) [58].

### 2.4. Outcome Measures

**Figure 1:**
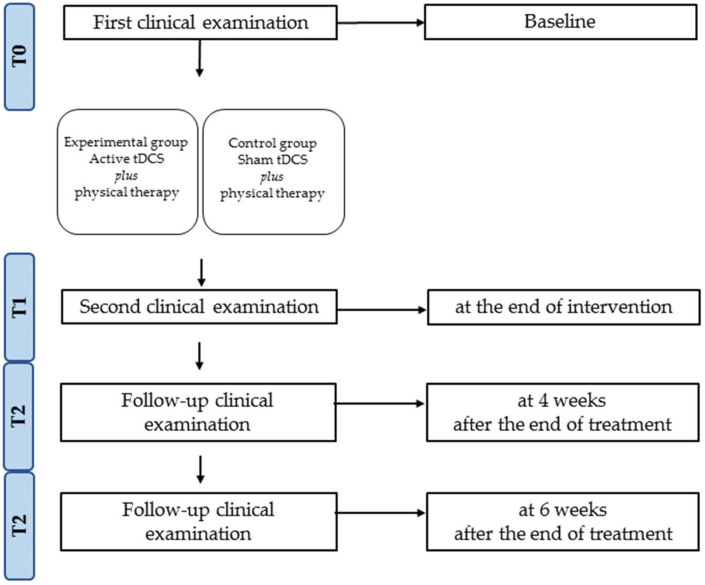
Study design and timeline.

The enrolled study participants were assessed at the baseline (T0), at the end of intervention (T1), at 4 weeks (T2) and 6 weeks after the end of treatment (T3), in terms of balance and gait, through TUG [59], 6MWT [60] (distance, velocity and cadence), Berg Balance Scale (BBS) and Fall Risk Index (FRI), obtained using the Biodex Balance System^®^ (as depicted by Figure 1).

In more detail, the 6MWT and TUG tests were measured using a single wearable inertial sensor (G-Sensor^®^ BTS Bioengineering S.p.A., Milan, Italy) to instrumentally confirm the time taken (seconds) in carrying out the TUG and the meters traveled during the 6MWT. This inertial sensor was positioned on the participant’s waist using a semi-elastic belt covering on the L1–L2 intervertebral disc for the TUG test and the L4–L5 region for walking assessment, providing acceleration values along three orthogonal axes. For the TUG assessment, participants were instructed to sit on a chair with back support and without armrests, then they stood up, walked for 3 m at self-selected speed, performed a 180° turn around at an obstacle, walked back to the chair and performed a second 180° turn to sit down [44]. The patient was also asked to perform a self-paced submaximal walk along a hard surface, trying to cover as much distance as possible and measuring the distance traveled for 6 min [61]. Each participant received no specific warm-up activity and a researcher walked to one side and behind the participant, close enough for safety purposes; the distance was recorded in meters [62]. Post-processing of the data using dedicated software (BTS G-Studio; BTS Bioengineering S.p.A.) allowed the following parameters to be computed: gait velocity and gait cycle obtained performing the 6MWT and TUG tests [63].

The BBS is a 14-item scale widely used to assess both dynamic and static balance disorders in MS, providing information about a patient’s balance-related abilities [64]. The BBS is scored on a 5-point ordinal scale (0 cannot perform–4 normal performance), with a higher score indicating better performance and with a maximum total score of 56. Lastly, to assess balance, this study used a commercially available balance device, the Biodex Balance System (Biodex Medical Systems, Shirley, NY, USA), to evaluate an individual’s ability to maintain dynamic postural stability [65]. The Biodex Balance System is a circular platform that moves freely and simultaneously about the anteroposterior and mediolateral axes. Patients were instructed to maintain the vertical projection with their center of gravity in the midpoint of the platform by observing a vertical screen located 30 cm in front of their face. Each assessment took 20 s, with 10 s rest periods in between. Patients stood barefoot on the platform with eyes open and the board was set to constant instability. The average of the results from 3 assessments was obtained as the normalized Fall Risk Index (FRI), a score obtained as the ratio between the FRI and the maximum predictive FRI for the relevant age, indicating a greater risk of fall than expected for that age (normalized FRI > 1) and those with an equal or even lesser risk of fall than expected for that age (Normalized FRI ≤ 1)[66].

### 2.5. Statistical Analysis

The collected data were analyzed using the statistical package R 3.5.2 (R foundation, Vienna, Austria). Descriptive analyses were generated for the demographic and clinical variables of the two arms. The normal distribution of the continuous variables was assessed via Shapiro–Wilk test. Because of the non-parametric distribution, a Mann–Whitney U test was performed to examine the effect of the intergroup treatment (active, sham) and the Wilcoxon test in an intra-group evaluation (pre-evaluation and postevaluation). The type I error (α) was set to 0.05, and the effect sizes were assessed using the rank biserial correlation (RBC). In addition, we conducted the Friedman test, a repeated measures statistical test of the ANOVA type, for non-parametric data. A post hoc evaluation was conducted to assess treatment or time point differences with an MFA and cluster evaluation.

To explore the dataset in more depth, revealing the existence of a difference at a certain time point, we used a factorial analysis and hierarchical clustering. An MFA is a multivariate data analysis method for summarizing and visualizing complex data, whereby individuals are described by several sets of variables [52,53,67]. An MFA takes into account the contributions of all variables to define the distances between individuals on a Cartesian chart through the proportion of variance (eigenvalues) of each variable attained by the different dimensions (axes) [68]. The variables with larger variance values contribute the most to the definition of the dimensions (axes) [69]. Thus, variables that contribute the most to dimension 1 (abscissa) and dimension 2 (ordinate) are the most important in explaining the variability and the position of individuals in the chart [70]. Given a graph of variables (correlation circle), it is possible to show the relationship between the variables and the dimensions [54,69,71] by computing the classical Euclidean distance from the principal coordinates. Lastly, we performed the MFA, a machine learning cluster method that weights the distances of participants influenced by variables, grouping individuals into clusters and computing the classical Euclidean distances from the principal coordinates [68,72]. We computed and visualized the multiple factor analysis in R software using FactoMineR (for the analysis) and fact extra (for data visualization).

To validate the MFA clustering, we performed K-means clustering as a machine learning algorithm, a model capable of dividing data in such a way that the degree of similarity between two data observations is maximal if they belong to the same group and minimal if they do not [73]. With the use of JASP v0.16 (JASP Team, Amsterdam, Netherlands), we obtained the R^2^, a score that indicates the amount of variance explained by the model; the Akaike Information Criterion (AIC) score, where lower values represent better clustering outputs; and the silhouette score, a value ranging from −1 to 1, where 1 represents dense and well-separated clusters [74].

## 3. Results

**Figure 2:**
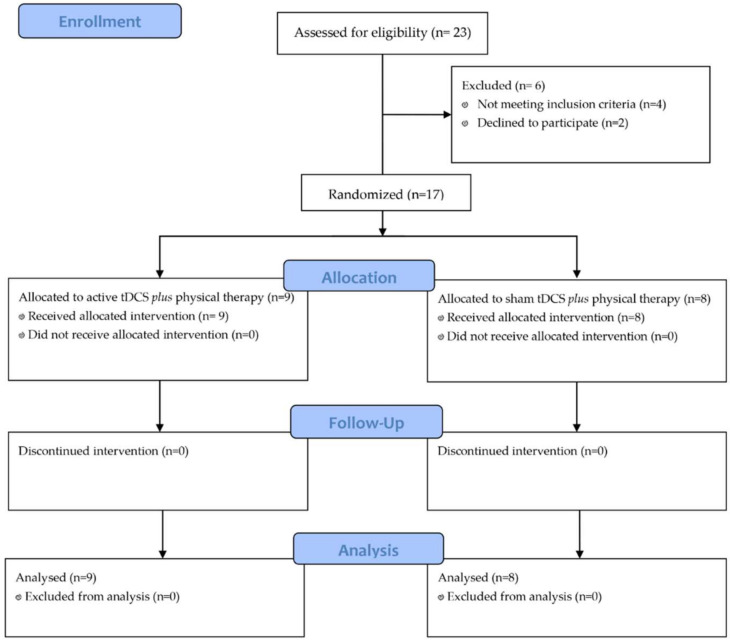
Study flow-chart.

Out of the 23 patients assessed for eligibility, 6 were excluded (4 did not meet the inclusion criteria for the diagnosis of secondary progressive MS, while 2 patients declined to participate). Seventeen patients were enrolled after providing informed consent and were randomized into two groups: tDCS plus physical therapy (n = 9), or the control group, undergoing sham tDCS and physical therapy (n = 8), as depicted in Figure 2.

There were no statistically significant differences between groups in terms of the demographic and clinical features at baseline (see Table 1 for further details).

Stimulation was well-tolerated across participants, with no relevant side effects for any participant. We demonstrated significant intra-group improvements for 6MWT in ΔT0-T1 (experimental group: *p* = 0.006; control group: *p* = 0.009), ∆T1-T2 (experimental group: *p* = 0.007; control group: *p* = 0.006) and ∆T2-T3 (experimental group: *p* = 0.006); for TUG in ∆T0-T1 (experimental group: *p* = 0.031; control group: *p* = 0.043) and ∆T2-T3 (experimental group: *p* = 0.042; control group: *p* = 0.045); and for BBS in ∆T0-T1 (experimental group: *p* = 0.023) (see Table 2).

However, the between-group analysis showed statistically significant differences in terms of gait (6MWT: RBC = 0.8; *p* = 0.003) and balance (BBS: RBC = 0.4; (*p* = 0.031) at the end of treatment (T1). Nevertheless, there were significant between-group differences in terms of the 6MWT scores, even at T2 (RBC = 0.93; *p* = 0.001) and T3 (RBC = 0.84; *p* = 0.007), with a positive but not significant difference between BBS and TUG at T2 and T3.

Subsequently, we performed a non-parametric repeated measures Friedman test and Kendall’s W test. The experimental group showed a significant difference in repeated measures compared to the control (experimental group: *p*-value = 0.03, W = 0.34 versus *p*-value = 0.15, W = 0.20). Moreover, we reported a significant decrease in gait velocity (m/s) in the control group (*p* = 0.01, W = 0.77), and a significant increase in gait cycle (meters) in the experimental group (*p* = 0.04, W = 0.31). Lastly, we demonstrated similar significant reductions in TUG (seconds) in both groups, as depicted in Table 3.

Given these positive results at the end of the treatment (T1), we assessed the benefits one month after the end of the tDCS application. Regardless of the intervention (active or sham), BBS and FRI did not change over time. Despite the Mann–Whitney U and rank biserial correlation calculations, we examined the weights of the variables at the various timepoints to understand how they could have changed over time with respect to the two groups.

**Figure 3:**
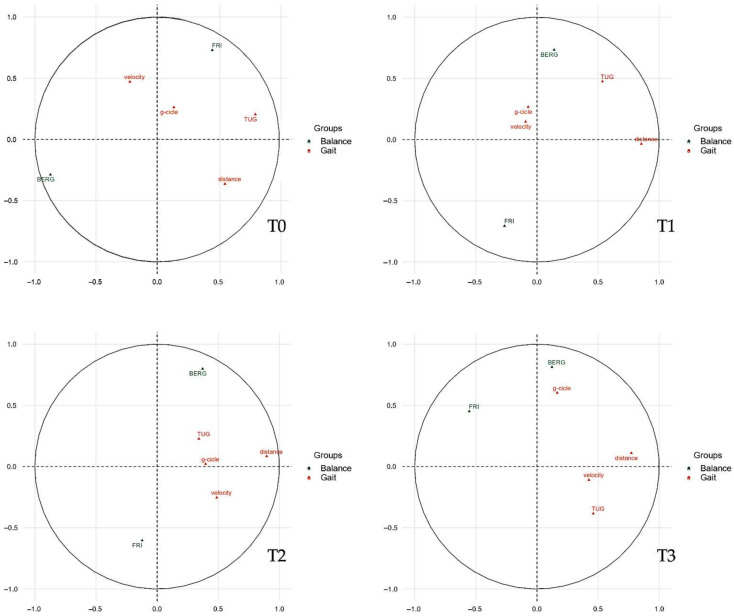
Correlations between quantitative variables and dimensions. The plot depicts the topographical influence in the arrangement of the variables on the graph along the abscissa (Dim1) and the ordinate (Dim2). Abbreviations: BERG: Berg Balance Score; Dim1: dimension 1 (abscissa axis); Dim2, dimension 2 (ordinate axis); g-cycle: gait cycle; TUG: timed up-and-go; FRI: Fall Risk Index.

As shown in Figure 3, we divided the parameters of balance (green) and gait (orange), then we evaluated the weights of the single variables through linear regression, representing them two-dimensionally on a Cartesian plane. Then, the variables were arranged as vectors based on their weight and proximity to the axes influencing the spatial positions of individuals at the various timepoints analyzed.

**Figure 4:**
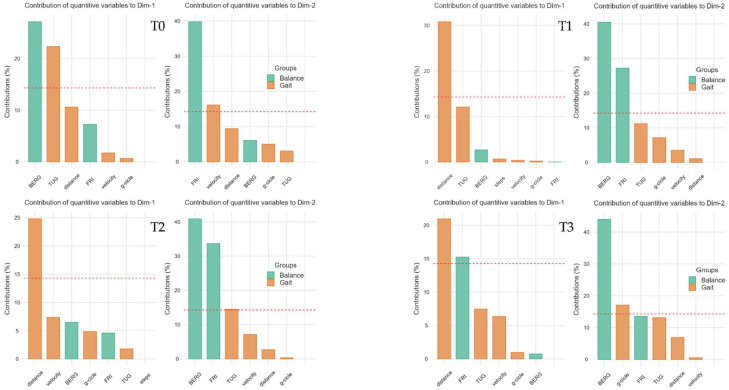
Contributions to the dimension graphs (Cartesian axes). The single weight of each variable in the construction of the Dim1 (abscissa axes of previous figure) and Dim2 (ordinate axes of previous figure) is graphed by bar plots. Abbreviations: BERG: Berg Balance Score; Dim1: dimension 1 (abscissa axis); Dim2, dimension 2 (ordinate axis); g-cycle: gait cycle; TUG: timed up-and-go; FRI: Fall Risk Index.

With Figure 4, it is possible to analyze the weight that each single variable gives to the dimension (Cartesian axes). In this scenario, it can be determined how a variable influences the participants in the study groups at each timepoint, conditioning the respective bidimensional position.

Finally, we analyzed the arrangement of individuals based on the weights and positions of the variables along the Cartesian axes (dimensions), as shown in Figure 4. The cluster arrangement of the two groups can help us to understand the weight and influence of the variables analyzed in the previous figures.

**Figure 5:**
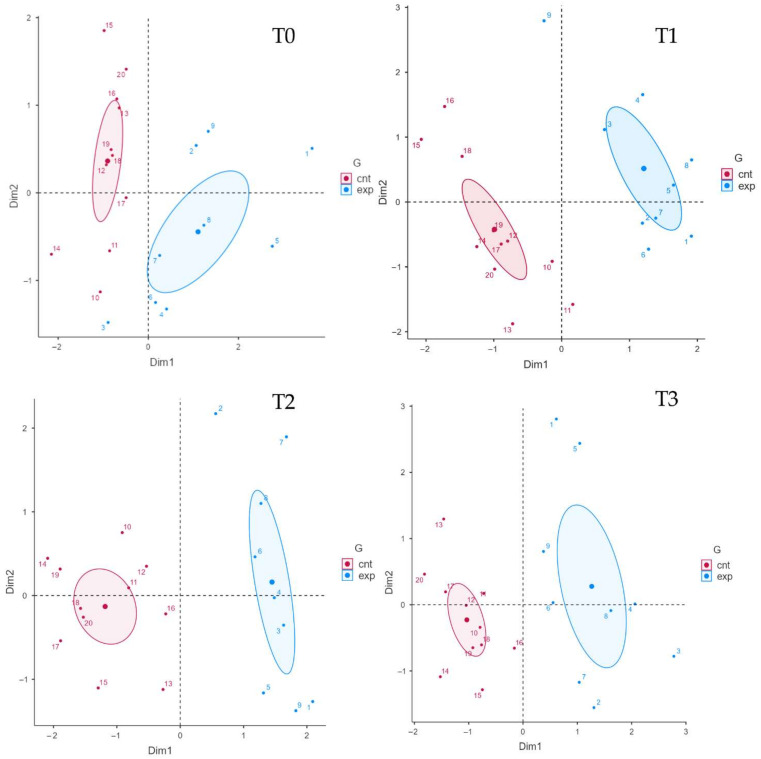
Clustered individual factors map. Each individual is positioned according to the Cartesian axes and thickens in specific clusters that reflect the influences of the dimension and each variable. Abbreviations: cnt: control group (sham tDCS + physical therapy); Dim1: dimension 1 (abscissa axis); exp: experimental group (real tDCS + physical therapy); G: groups.

In this scenario, the individuals of the experimental group with active tDCS are placed in the upper right corner, clearly detaching the participants of the control group at the end of the treatment (T1). This characteristic is due to the positive influence of the “distance” variable on the abscissa axis (dimension 1, Dim1) and the positive correlation of the BBS on the ordinates (dimension 2, Dim2). The cluster representation is confirmed with a similar arrangement in T2, and partially due to the influence of the gait outcome and the correlations of balance variables, keeping the experimental cluster in the right upper quadrant detached from the control cluster, as in T1 (for further details see Figure 5).

We assessed the quality of clustering by adapting the data to a K-means machine learning approach, reporting an observed R^2^ value of 0.54, demonstrating that the model shows good reliability; an AIC of 976.45, which demonstrate a moderate quality of fit of the model with several clusters; and a Silhouette Index of 0.34, with a low overlap trend for a small sample.

## 4. Discussion

The present pilot RCT suggests the potential enhancement of tDCS when supplemented with physical exercise in improving gait and balance in MS patients in the short term.

Our findings reported for the experimental group significant improvements in 6MWT scores for all time-points (∆T0-T1: *p* = 0.006; ∆T1-T2: *p* = 0.007; ∆T2-T3: *p* = 0.006), for TUG in ∆T0-T1 (*p* = 0.031) and ∆T2-T3 (*p* = 0.042) and for BBS in ∆T0-T1. The main finding is positive results of the 6MWT in the active intervention, considering that TUG scores significantly changed regardless of the intervention. There were statistically significant differences in 6MWT (*p* = 0.003) and BBS (*p* = 0.031) between groups at the end of treatment (T1). Furthermore, there were also significant between-group differences in terms of 6MWT scores at T2 (*p* = 0.001) and T3 (*p* = 0.007).

These data were further considered after performing a cluster analysis; considering the weights of each parameter measured, the variables related to walking and balance were similarly disposed both at the end and at 4 weeks after the end of treatment. This disposition might indicate a prolonged beneficial effect induced by the combination of these rehabilitative therapies.

We shed light on the presence and persistence of the effects induced by the proposed treatment, considering the therapeutic exercise as a reference and estimating the modulatory effects over time driven by the addition of tDCS. The statistical analysis showed that exercise interventions moderately improved the walking distances immediately after the end of treatment, losing effect 4 and 6 weeks later. The addition of tDCS appeared to provide an ameliorative effect, with significant increases in the distances walked by patients in the experimental group at T1, as in a recent review [35], and significant increases in BBS for people with MS [75]. At 4 weeks after the end of the treatment, we found a loss of gain compared to T1, considering that it was significantly lower in the experimental group than in the control group. Indeed, in this latter group, the drop in the number of meters covered increased as time progressed, with a discontinuation of the beneficial effect that was previously appreciated. The scientific literature defines therapeutic exercise as the treatment of choice for gait disorders, allowing improvements in autonomy, mobility and consequently quality of life in patients with MS [17,76]. Systematic reviews and meta-analyses have shown small but clinically meaningful effects for exercise training in terms of walking distance, speed, endurance and stride length, being homogeneous across modes of exercise training (i.e., aerobic vs. resistance) [77,78,79]. The effects of exercise appear to require prolonged treatment over time, from 3 weeks to 24 weeks, resulting in significant changes in gait parameters measurable from 12 weeks of treatment onward [80,81]. It is not known how long the advantages acquired by a conventional treatment will last, in light of which a correct therapeutic prescription could be defined. In the present study, a daily treatment shortened in time was able to bring a small gain in walking distance that was not maintained over time, thereby indicating the need to repeat the treatment protocol or to identify a combination treatment that can prolong the effects over time. This is what seemed to have happened in the experimental group, in which the initial benefit was only partially reduced over time, with a return to the baseline condition only 6 weeks after the end of treatment.

The stimulation effect appears to be based on the neuromodulating effect that a direct current of low intensity is able to determine on cortical activity [32]. Stimulating the hemisphere contralateral to the most affected limb, we targeted a cortical region in which neuroplasticity appears to be more difficult to recover, as demonstrated by the work by Chaves, et al. [82]. Exercise alone appears to have less neuroplastic potential on the hemisphere contralateral to the weaker limb [83]. Considering that walking is a complex motor task that requires properly balanced activation of the lower limbs, the addition of a neuromodulating treatment improved the motor performance of the most affected limb by improving the motor task overall.

Furthermore, a protocol of 10 sessions of stimulation associated with gait training represents the current reference, since it is clear that a small number of sessions has proven to be ineffective [34,35,36]. The immediate impact of tDCS is now well documented [40], although what is not well investigated is the persistence of this effect after the end of the stimulation itself. A recent study by Pilloni, et al. [36] shows that multiple sessions of tDCS coupled with aerobic exercise lead to persistent improvements in distance covered by performing the 2MWT, indicating a lack of effect determined by exercise alone. In our pilot RCT, the effect of exercise alone on 6MWT distance is present at the end of treatment and returns to baseline conditions as early as 4 weeks after the end of treatment. The difference between the studies could be due to the different MS patient populations (RR vs. RR + SP) and the degree of EDSS. In a previous study, Savci, et al. [84] included MS patients with a median EDSS score of 4.0 (range 1.5 to 6.0), with longer distances performed in the 6MWT compared to the results reported in our study. This can be explained by the use of median and IQR data in a frequency distribution with a number of extreme values, which among other things pushes the curve towards a lower EDSS score.

In patients with MS, the TUG execution provides additional details on functional mobility and independence, measuring the ability to sit and stand up from a chair, walk in a straight line and change direction in an easy and comfortable way [85,86]. Both the control and experimental groups improved their ambulatory functional performances, with reductions in the time required to perform the TUG test, keeping the results obtained at the end of the treatments stable even at 6 weeks. These results have a positive impact on the patient’s quality of life, being closely associated with the demands and activities of daily living.

The synergy of tDCS and exercise can not only improve motor performance, but also can positively affect postural control, balance and fall risk [87]. Considering the close relationship between walking and balance control, patients underwent a specific rehabilitation protocol. A clinical balance assessment using the BBS revealed an important improvement of the total score in the experimental group, which was not recorded in the control group. Indeed, the control group subjected to sham stimulation in addition to balance training recorded a non-significant improvement of only one point, maintained one month after the end of treatment. This short-lived effect was lost 6 weeks after the completion of treatment. In contrast, the active stimulation resulted in a 5-point gain at the end of treatment and a modest gain at one month, with a loss of effect at 6 weeks. These are encouraging results, considering that a 3-point change in the BBS is the cut-off score to correctly classify a clinically important change in balance performance during activities of daily living [64].

In this pilot study, we showed that tDCS might add positive effects when combined with physical therapy in terms of balance and gait, albeit these findings could not be seen after 4 weeks. However, the machine learning approach, through the analysis of the clusters between the beginning and end of treatment, underlined an influence on BBS in the experimental group. In more detail, the combination of tDCS and physical therapy appeared to maintain the direction of arrangement on the Cartesian axes at T2 in the MS patients in the study group. These findings were not properly in line with the classic statistical analysis, which did not show a significant difference between groups at 4 weeks after the end of treatment (T2). In this scenario, the multiple factor analysis could legitimize the improvements in balance with a common vectorization of the study clusters, in the sense of improvements in BBS, Fall Risk Index and walking performance scores in the active tDCS group compared to the sham tDCS group.

At T3, the improvements appeared to decrease, although the parameters of balance and gait that did not seem to carry over to T2 with an intergroup analysis, as could be found through a machine learning analysis. In fact, through an evaluation of the grouping of the clusters based on the outcomes, the results obtained at the end of the treatment seemed to be similar even 6 weeks after the end of the intervention.

We are aware that our study has some limitations. First, the sample size could be considered relatively small for a machine learning model, although in addition to strict eligibility criteria, it should also be considered that we chose a cluster approach that provides an analysis of the arrangement of groups based on the variables entered, excluding predictive analyses. Second, any drop in measurement quality can prevent machine learning algorithms from accurately modeling the non-linear associations between features. However, it should be noted that we have estimated almost two-thirds of the variance for the two dimensions. Third, in the context of the results obtained, we considered the addition of an intervention that is not always accessible, and which requires skills in execution. Lastly, several tDCS studies in MS have focused on fatigue, although this variable was been included in the present work due to the already in-depth potential of prefrontal area stimulation [88,89,90,91,92,93].

On the other hand, to the best of our knowledge, this is first study that analyzed the effects of tDCS combined to physical therapy in terms of balance and gait outcomes in patients with MS, not only through a standard statistical analysis but also through a machine learning approach.

## 5. Conclusions

Taken together, the present pilot RCT showed that tDCS coupled with physical therapy might have a significant effect on 6MWT in MS patients. This treatment seemed to not maintain the improvements on balance at one month after the end of treatment, although a similar distribution through the weight of gait and balance outcomes was shown by the cluster-type machine learning analysis. These promising findings might indicate a prolonged beneficial effect induced by the combination of these rehabilitative therapies, although perspectives will be required to consider task-related stimulations, perhaps using coupling a virtual reality approach. Further research is necessary to investigate the efficacy of novel rehabilitative interventions such as tDCS in patients affected by MS, also considering the use of a machine learning approach.

## Figures and Tables

**Table 1:** Baseline characteristics of the experimental group (tDCS plus physical therapy) and control group (sham tDCS plus physical therapy).

	Experimental Group (n = 9)	Control Group (n = 8)	*p* Value
Male/Female	3/6	2/6	*p* = 0.213
Age (years)	43.22 ± 10.46	39.75 ± 8.39	*p* = 0.081
EDSS	3 [1]	3 [0]	*p* = 0.154
6MWT (meters)	300.1 ± 18.8	294.5 ± 13.39	*p* = 0.134
Gait velocity (m/s)	0.87 ± 0.26	0.92 ± 0.24	*p* = 0.070
Gait cycle (meters)	1.01 ± 0.17	0.96 ± 0.46	*p* = 0.076
TUG (seconds)	14.11 ± 3.5	12.8 ± 1.1	*p* = 0.072
BBS	48.33 ± 7.4	50.4 ± 1.4	*p* = 0.356
FRI	1.31 ± 0.3	1.25 ± 0.3	*p* = 0.069

Continuous variables and parametric data are expressed as means ± standard deviations; categorical variables are expressed as counts (percentages) or medians (interquartile ranges) for non-parametric data; ratios are expressed as x/y. Respectively, we performed the X^2^ and Mann–Whitney U tests. Abbreviations: 6MWT: 6 min walking test; BBS: Berg Balance Scale; EDSS: Expanded Disability Status Scale; FRI: Fall Risk Index; tDCS: transcranial direct current stimulation; TUG: timed up-and-go.

**Table 2:** Intra-group differences in the outcome measures for experimental (tDCS plus physical therapy) and control (sham tDCS plus physical therapy) groups.

		T0	T1	∆T0-T1 *p*-Value	T2	∆T1-T2 *p*-Value	T3	∆T2-T3 *p*-Value
6MWT (meters)	*experimental*	300.1 ± 18.8	322.0 ± 17.0	**0.006**	317.3 ± 19.1	**0.007**	303.7 ± 16.9	**0.006**
	*control*	294.5 ± 13.4	299.5 ± 14.5	**0.009**	284.2 ± 14.9	**0.006**	281.5 ± 13.7	0.084
Gait velocity (m/s)	*experimental*	0.9 ± 0.3	0.8 ± 0.3	0.331	0.8 ± 0.5	0.105	0.8 ± 0.5	0.312
	*control*	0.9 ± 0.2	0.9 ± 0.4	0.227	0.8 ± 1.2	0.109	0.7 ± 0.4	0.059
Gait cycle (meters)	*experimental*	1.0 ± 0.2	0.8 ± 0.4	0.451	0.8 ± 0.41	0.245	0.8 ± 0.2	0.677
	*control*	1.0 ± 0.5	1.1 ± 0.4	0.423	1.0 ± 0.52	0.311	0.9 ± 0.5	0.546
TUG (seconds)	*experimental*	14.1 ± 3.5	12.8 ± 5.0	**0.031**	13.0 ± 3.7	0.154	12.0 ± 3.9	**0.042**
	*control*	12.8 ± 1.1	11.3 ± 3.9	**0.043**	10.3 ± 2.6	0.06	11.3 ± 2.1	**0.044**
BBS	*experimental*	48.3 ± 7.4	53.3 ± 3.9	**0.023**	52.2 ± 5.1	0.095	50.3 ± 6.8	0.083
	*control*	50.4 ± 1.4	51.4 ± 4.4	0.162	52.4 ± 3.3	0.094	49.8 ± 2.0	0.079
FRI	*experimental*	1.3 ± 0.3	1.0 ± 0.8	0.099	1.0 ± 0.6	0.876	1.0 ± 0.5	0.765
	*control*	1.3 ± 0.3	1.2 ± 0.5	0.203	1.2 ± 0.6	0.785	0.1 ± 0.6	0.672

Continuous variables are expressed as means ± standard deviations. Wilcoxon’s paired t-Test was used for intra-group differences. Note: *p*-values are significant if <0.05 (expressed in bold type). Abbreviations: 6MWT: 6 min walking test; BBS: Berg Balance Scale; FRI: Fall Risk Index; tDCS: transcranial direct current stimulation; TUG: timed up-and-go.

**Table 3:** Repeated measure differences (Friedman test) in the outcome measures for the experimental (tDCS plus physical therapy) and control (sham tDCS plus physical therapy) groups.

		Chi-Squared	*p*-Value	Kendall’s W
6MWT	experimental	9.14	**0.03**	0.34
	control	5.35	0.15	0.20
	between-group	9.24	**0.03**	0.23
Gait velocity	experimental	2.00	0.57	0.07
	control	20.92	**0.01**	0.77
	between-group	2.43	0.49	0.34
Gait cycle	experimental	8.43	**0.04**	0.31
	control	1.25	0.74	0.05
	between-group	0.60	0.90	0.05
TUG	experimental	9.93	**0.02**	0.37
	control	11.4	**0.01**	0.42
	between-group	5.64	0.13	0.35
BBS	experimental	4.48	0.21	0.17
	control	3.32	0.34	0.12
	between-group	3.74	0.29	010
FRI	experimental	4.74	0.19	0.18
	control	4.75	0.19	0.18
	between-group	3.24	0.36	0.12

Note: *p*-values are significant if <0.05 (expressed in bold type). Abbreviations: 6MWT: 6 min walking test; BBS: Berg Balance Scale; FRI: Fall Risk Index; tDCS: transcranial direct current stimulation; TUG: timed up-and-go.

## Data Availability

Not applicable.
